# Alzheimer's Disease: Another Target for Heparin Therapy

**DOI:** 10.1100/tsw.2009.100

**Published:** 2009-09-01

**Authors:** Luigi Bergamaschini, Emanuela Rossi, Carlo Vergani, Maria Grazia De Simoni

**Affiliations:** ^1^ Department of Internal Medicine, Geriatric Unit, Ospedale Maggiore Policlinico IRCCS, University of Milan, Italy; ^2^ Department of Neuroscience, Mario Negri Institute for Pharmacological Research, Milan, Italy

**Keywords:** Alzheimer’s disease, heparin, inflammation, amyloid-beta, complement system, contact/kinin system

## Abstract

Alzheimer's disease (AD) is the leading cause of dementia and cognitive decline in the elderly. Brain tissue changes indicate that the two main proteins involved in AD are amyloid-β(A-β), which is associated with the formation of senile amyloid plaques, and tau, which is associated with the formation of neurofibrillary tangles. Although a central role for A-β in the pathogenesis of AD is indisputable, considerable evidence indicates that A-β production is not the sole culprit in AD pathology. AD is also accompanied by an inflammatory response that contributes to irreversible changes in neuronal viability and brain function, and accumulating evidence supports the pivotal role of complement and contact systems in its pathogenesis and progression. The complexity of AD pathology provides numerous potential targets for therapeutic interventions. Compounds that interact directly with A-β protein or interfere with its production and/or aggregation can reduce the inflammatory and neurotoxic effects of A-β, and heparin, a glycosaminoglycan mixture currently used in the prophylaxis and treatment of thrombosis, might be a candidate, as recent research has been extended to consider its nonanticoagulant properties, including its modulation of various proteases and anti-inflammatory activity.

## INTRODUCTION

Alzheimer's disease (AD), the main cause of dementia in the elderly, is becoming an ever-increasing problem as the population ages[1,2,3]. Its basic pathological mechanism is represented by conformational changes in amyloid-β peptides (Aβ) and tau proteins, two normally expressed proteins that self-assemble into toxic β-pleated sheet aggregates, and its main histopathological features are neuritic plaques formed by the extracellular deposition of Aβ and neurofibrillary tangles, which consist of intracellular aggregates of hyperphosphorylated tau proteins in the cytoplasm of neurons[4,5].

Normal tau promotes assembly and stabilized microtubules, but the nonfibrillized, abnormally hyperphosphorylated form sequesters normal tau and disrupts microtubules. The abnormal hyperphosphorylation also promotes misfolding, a decreased turnover, and self-assembly into tangles of paired helical or straight filaments[6]. Microtubule disruption and the aggregation of tau in neurofibrillary tangles probably impairs axoplasmic flow and leads to the slowly progressive retrograde degeneration and loss of connectivity of the affected neurons[7].

Aβ in neuritic plaques is a 39-43 residue peptide that is a cleavage product of the amyloid precursor protein (APP)[8,9]. Most physiological fluids, such as plasma and cerebral spinal fluid (CSF), contain derivatives of APP, including water-soluble Aβ peptides. The aggregation of these monomeric Aβ peptides into oligomeric forms is associated with conformational changes and neurotoxicity in AD[10,11,12], although it is still not known whether this aggregation and the deposition of the oligomers in plaques are steps in the same pathway[13].

It is thought that apolipoprotein E (ApoE, and especially its ε4 isoform), α1-antichymotrypsin, and C1q complement factor increase the formation of Aβ fibrils from water-soluble Aβ, and promote and stabilize the transformation[14,15,16,17,18]. It is also thought that one critical event in the pathological mechanism of AD is the reaching of a crucial concentration of water-soluble Aβ or chaperone proteins in the brain, at which point the conformational changes lead to the formation of aggregates and thus initiate a neurodegenerative cascade. In the case of sporadic AD, this crucial concentration might be reached because of any combination of the age-associated, over-production of Aβ, impaired brain clearance, and the influx of circulatory Aβ into the central nervous system[19].

Given the central role of Aβ in the pathogenesis of AD, research in the last decade was aimed at developing therapies that target amyloid production, aggregation, clearance, or toxicity[20,21,22, 23,24,25]. In this area, β-secretase inhibitors[26,27,28,29,30], statins[31,32,33,34,35], and Aβ vaccination procedures[36,37,38,39,40] that aimed to inhibit Aβ production or accumulation appear to be some of the more promising approaches.

However, although the Aβ cascade hypothesis is one of the prevalent theories[41], some researchers suggest that this accumulation may not be sufficient to cause cognitive impairment[42], but it is necessary to trigger a series of other events (including the up-regulation of inflammatory responses, tau processing, and changes in free radicals) that cause self-perpetuating brain injury[43,44]. There is strong evidence that inflammation is characteristic of AD[45] in human beings and animal models: the senile plaques are decorated by a number of inflammatory proteins, complement and kinin system factors, and Aβ deposition is accompanied by the attraction and activation of microglia and astrocytes, which leads to increased secretions of proinflammatory cytokines as part of a neuroinflammatory response[20].

Although the mechanism by which Aβ induces toxicity is still poorly understood, it is possible that therapies that target Aβ toxicity, such as modulation of the Aβ-mediated inflammatory reaction, will be developed. Given their many effects, heparins and heparin-related compounds could be valuable therapeutic candidates[46,47,48,49,50,51,52,53,54,55,56].

## MULTIPLE FUNCTIONS OF HEPARIN

Heparin, a linear sulfated polysaccharide chain, belongs to the family of glycosaminoglycans (GAGs) that play important roles in blood clotting, neuronal communication, brain development, and cancer by binding to different proteins[57,58]. GAG families have different molecular weights, charge densities, degrees of sulfation, and types of disaccharide units, which create an enormous number of protein-binding motifs and are responsible for their biological activities[59].

GAGs have been considered of interest in AD ever since they were first demonstrated in amyloid plaques and neurofibrillary tangles. *In vitro*, heparan sulfate proteoglycans (HSPGs) may regulate APP processing by Alzheimer's β-secretase and may enhance Aβ fibrillogenesis, but they may also prevent the persistence of the toxic forms of Aβ oligomers or protofibrils by transforming them into more harmless aggregates[21], and proteoglycan analogue dextran sulfate and chondroitin sulfate may modulate the activation of the classical and alternative complement pathways[60,61,62,63]. The precise role of GAGs in AD is, therefore, still controversial.

Heparin is a highly sulfated GAG consisting of hexuronic acid and D-glucosamine residues joined by glycoside linkages[64]. Its most widely accepted function is as an anticoagulant and antithrombotic agent, mainly due to its ability to potentiate antithrombin III activity[65,66]. The early studies of heparin structure and function concentrated on the interactions responsible for its inhibition of blood coagulation, but the observation that heparin-containing tissues are in direct contact with the external environment suggests it may also play a role in host defense. Heparin binds a number of growth factors[67], as well as extracellular matrix proteins[68,69,70,71], proteins involved in lipid metabolism (such as apolipoprotein)[72,73,74], and acute phase[75,76,77,78,79,80,81] and complement factors[82,83,84,85, 86,87]. There is also accumulating evidence that it interferes with the adhesion of leukocytes to the endothelium, a mechanism that plays a central role in the inflammatory response.

Furthermore, heparin may also be involved in regulating apoptosis[64,88]. There is evidence that it can modulate the activity of tumor necrosis factor (TNF) and nuclear factor kappa B (NF-kB), two key members of the apoptotic cascade; it can inhibit the enzymes responsible for DNA fragmentation in apoptotic cells[89]; and it can bind with high affinity to leukocyte receptors during apoptosis[90].

## POSSIBLE BENEFICIAL ACTIONS OF GAGS AND HEPARIN IN AD

Proteins undergo various types of post-translational modifications in cells under normal and stressed conditions. In some cases, the modified protein has reduced conformational stability and, therefore, an increased propensity to misfold and aggregate. One of the post-translational modifications that promote the aggregation of some proteins is proteolysis, and the most intensively studied example of a fibrillogenic polypeptide generated by proteolysis is the amyloid peptide.

Evidence that Aβ accumulation probably contributes to AD provides the rationale for a therapy based on altering brain Aβ accumulation and reducing its cytotoxic and proinflammatory action. The Aβ peptide is generated from APP: APP is initially cleaved by β-secretase, which is pivotal for the subsequent cleavage of APP fragments by γ-secretase that leads to the long fibrillar Aβ1-40 and Aβ1-42 peptides (Fig. 1). In AD, these peptides aggregate to form plaques within the brain. The protease responsible for β-secretase activity in neurons has been identified as β-site APP-cleaving enzyme 1 (BACE-1)[91], whose activity is crucial to the amyloidogenic processing of APP. An interesting recent study showed increased BACE-1 protein and enzymatic activity within AD brain homogenates, thus further supporting the hypothesis that abnormal BACE-1 activity is associated with the disease. When assessing the *in vivo* CSF concentration of BACE-1 and its activity, the authors found increased levels of both in subjects with MCI (mild cognitive impairment) in comparison with healthy controls and AD patients, thus demonstrating the early presence of abnormal BACE-1 concentrations in a group at risk for AD[92].

It is known that heparin and heparan sulfate regulate the activity of a number of proteases, and it has been reported that heparin inhibits BACE-1 activity *in vitro*[93]. It has been found that a low concentration of heparin can stimulate recombinant human BACE-1 activity *in vitro*, and stimulation by heparin leads to increased autocatalysis cleavage of the protease domain and a subsequent loss of enzyme activity[46]. Patey et al. evaluated engineered heparin analogues (including modifications designed to increase bioavailability) as novel BACE-1 inhibitors *in vitro* and found that a number of the tested compounds were effective; they also provided insights into the structural interaction between BACE-1 and heparin, indicating that the structure of the polysaccharide is much more important than its charge[56].

*In vitro*, charged residues within the 1-11 region are critical for Aβ proinflammatory activity[94,95,96]; thus, inhibiting this activity by pharmacologically targeting this region may also be useful in slowing the progression of neurodegeneration in the AD brain. One candidate for such a therapy is heparin□[50,52,53,54].

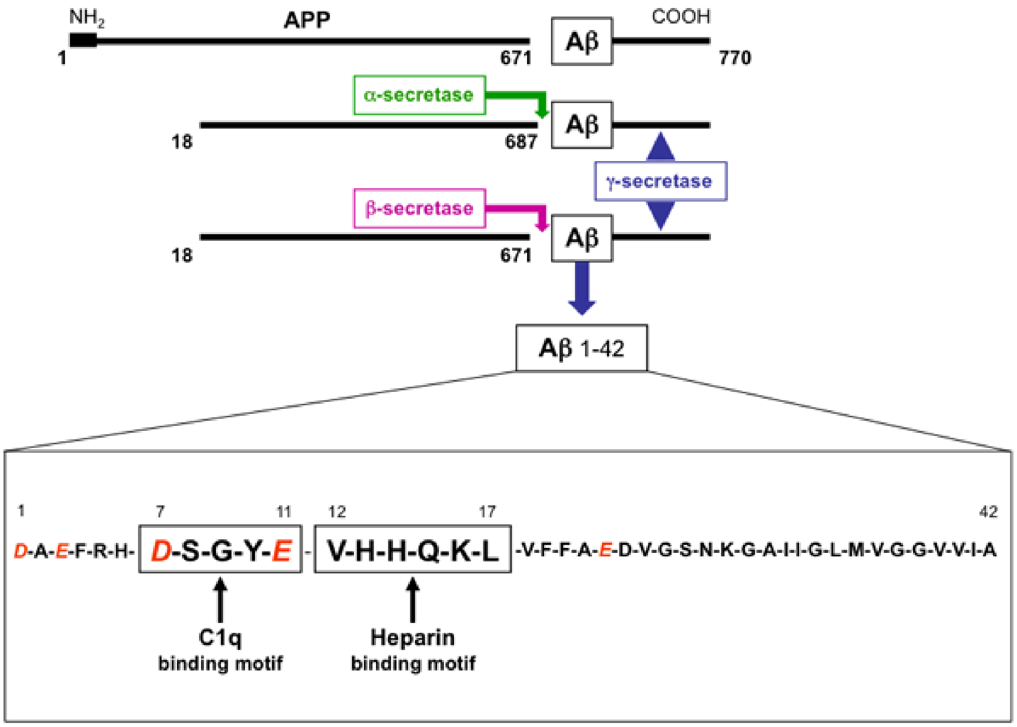

**FIGURE 1.** Amino acid sequence of Alzheimer's Aβ, the 39-43 residue peptide derived from proteolytic cleavage of the APP. Negatively charged residues are in red. The binding site of heparin (residues 12-17) could give place to a steric hindrance to the negatively charged residues within the region (residues 1-11) that is critical for the activation of complement and contact/kinin systems.

The heparin binding site on Aβ is the 13-16 region (HHQK) of the Aβ peptide, and represents the only target for the prevention of Aβ fibril formation. Heparin can prevent more than 70% of its binding to HSPGs and may block the cell surface adherence of Aβ. Either effect could protect neurons and vascular endothelial cells against the toxic effects of Aβ. It has been demonstrated that GAGs and other sulfate-containing compounds significantly attenuate the toxic effect of Aβ on neuronal differentiated PC12 cells[48,97,98,99], and their association with Aβ may prevent aggregation from occurring or induce aggregation of a different kind from that producing the intermolecularly stacked β-pleated sheet aggregates characteristic of the toxic form of Aβ. Alternatively, they might coat the aggregated Aβ in such a way that it cannot interact efficiently with cells to produce its toxic response or displace its interactions with cell-surface HSPGs.

## HEPARIN AND METAL CATIONS

Aβ is a metalloprotein that possesses a selective high- and low-affinity metal binding site[100,101]. Both ionic zinc and copper accelerate the aggregation of Aβ and promote release of reactive oxygen species from cells[102,103,104,105,106,107,108]. The AD brain seems to be characterized by elevated iron levels and an accumulation of copper ($Cu^{2+}$) and zinc ($Zn^{2+}$) in the hippocampus, the region of the AD brain more severely affected, in the CSF, and senile plaques[109,110]. In 1994, Bush and Tanzi discovered for the first time that Aβ becomes amyloidogenic in reaction to stoichiometric amounts of $Zn^{2+}$ and $Cu^{2+}$[111,112], and recently they coined "The Metal Hypothesis of AD", which proposed that the interaction of Aβ with these two metals drives Aβ pathogenicity and downstream AD pathology[113].

Classically, $Zn^{2+}$ and $Cu^{2+}$ ions bind heparin, modulating its anticoagulant activity[114,115,116]. Nevertheless, heparin might act positively in AD by binding these cations and thus reducing their unwanted activities, namely on APP physiology and Aβ aggregation[102,111,112,117]. It has been shown recently that heparin could also have an important role in the modulation of the activity of the extracellular superoxide dismutase (SOD)[118]. SOD3, a homotetrameric $Cu^{2+}$- and $Zn^{2+}$-containing glycoprotein, plays multiple roles[119,120,121,122], including retention of memory[123]. The importance of heparin in SOD3 activity is suggested by the increased risk of myocardial infarctions and stroke in individuals that carry a common polymorphism, R213G, which reduces the heparin affinity of SOD3[124].

## HEPARIN AND ITS ANTI-INFLAMMATORY ACTION

One of the downstream events involved in AD is a chronic inflammatory response. Over the last 20 years, a wide range of inflammatory markers that are not usually found in normal elderly subjects have been reported in AD patients[125] and increasing evidence suggests that sustained brain inflammation may be an essential cofactor in the pathogenesis of AD[126,127]. In addition to its direct cytotoxic effect, Aβ triggers a local inflammatory reaction that stimulates astrocytes, microglia, and cerebral vascular endothelial cells to produce a variety of inflammatory mediators, including cytokines and superoxide radicals that may be responsible for the chronic neurodegeneration. There is growing evidence to support the pivotal role of two inflammatory cascades in the pathogenesis or progression of AD: the complement and contact/kinin systems[94,127,128,129](Fig. 2).

### The Complement System

The complement system forms part of the innate immune system and has three major physiological activities: to defend against bacterial infection, to bring together innate and adaptive immunity, and to clear immune complexes and the products of inflammatory injury. As a key mediator of inflammation, complement contributes to tissue damage in various clinical disorders. It is activated by means of three pathways: the classical antibody-dependent pathway, the alternative pathway, and the lectin pathway leading to the formation of the cytolytic membrane attack complex C5b-9 (MAC)[130,131]. After complement activation, the biologically active peptides C5a and C3a elicit a number of proinflammatory effects, such as chemotaxis; the degranulation of phagocytic cells, mast cells, and basophils; smooth muscle contraction; and increased vascular permeability. Upon cell activation by these complement products, the inflammatory response is further amplified by the subsequent generation of toxic oxygen radicals, and the induction of the synthesis and release of arachidonic acid metabolites and cytokines[132,133].

Under physiological conditions, complement activation is effectively controlled by the action of a number of soluble inhibitors (C1-inhibitor [C1-INH], C4 binding protein, factors H and I, S-protein [vitronectin and clusterin]) and surface proteins (complement receptor 1, the membrane cofactor protein [MCP, CD46], decay-accelerating factor, the C8-binding protein, and CD59)[134].

Clinical and experimental evidence underlines the major role of complement in the pathogenesis of numerous inflammatory diseases, including neurodegenerative disorders such as AD, multiple sclerosis, and Guillain-Barré syndrome[45,135,136,137,138,139,140,141,142,143,144,145,146,147,148].

In the brain of AD patients, complement factors such as C1q, C4, C3, factor B, and C5b-9 have been found to colocalize with amyloid plaques and vascular amyloid in the cerebral cortex and hippocampus[149,150,151], as well as with myelin and membranes[152,153,154]. *In vitro*, Aβ fibrils activate the classical complement pathway by directly binding to C1q[95,96,152,153,155, 156,157,158,159,160] and the alternative pathway via interactions with C3[54,161].

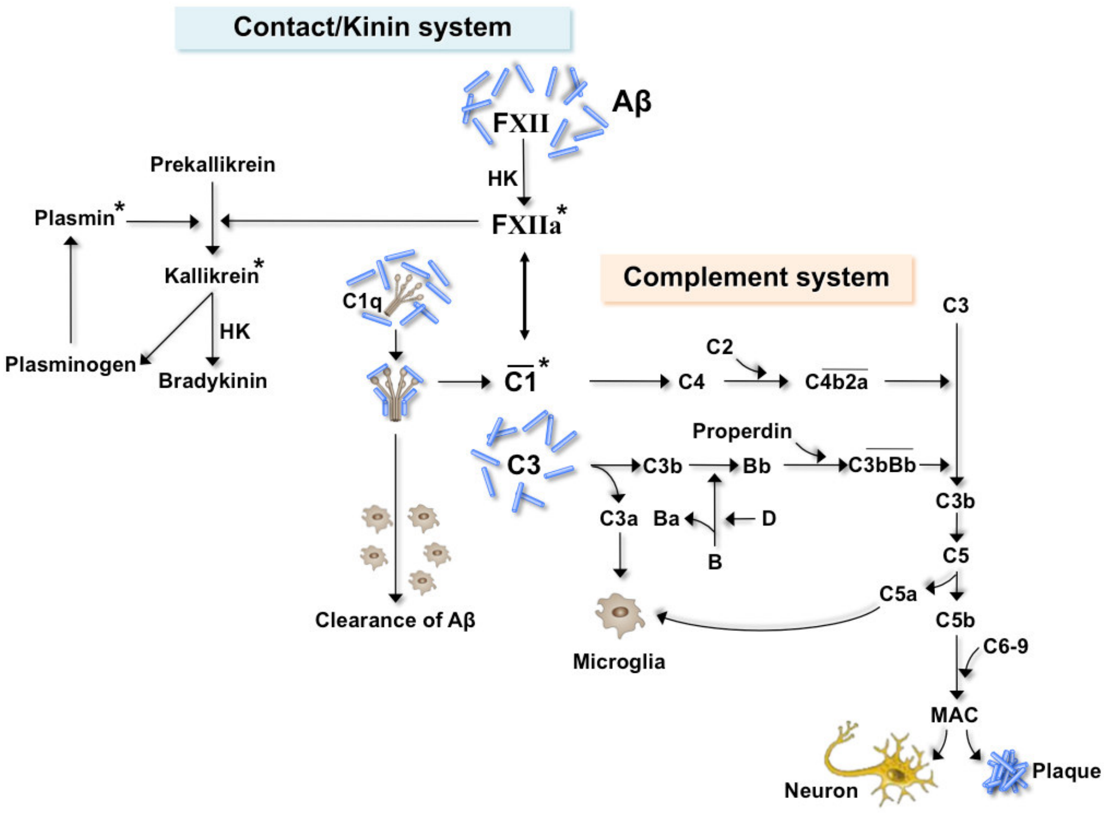

**FIGURE 2.** Schematic representation of the Aβ-dependent activation of complement and contact/kinin systems. Activation of contact system can lead to a C1q-independent activation of the complement classical pathway. Complement and contact/kinin systems share C1-INH as a major inhibitor (* = sites of action). In the AD brain, complement (both classical and alternative pathways) could induce both a detrimental effect (C3, MAC deposition on plaques, recruitment and activation of microglia, neurodegeneration) and neuroprotection (clearance of Aβ fibrils, promotion of neurogenesis), according to the degree of its activation.

Complement activation is common in AD, but its contribution to the pathogenesis of the disease is controversial[134]. It has been suggested that it may protect against Aβ-induced neurotoxicity and even contribute to reducing the accumulation of Aβ in senile plaques. By increasing the efficiency of glial phagocytosis, C1q binding to fibrillar Aβ and the subsequent formation of the C4 and C3 opsonins, and C3a and C5a chemoattractant anaphylatoxins, may accelerate Aβ clearance and thus reduce its deposition. In transgenic animals, complement inhibition leads to increased AD, whereas increased C3 production reduces Aβ deposition[162]. However, complement activation may also lead to neurodegeneration. Aβ-dependent complement activation induces microglial activation, thus leading to the secretion of proinflammatory cytokines and further Aβ generation, both of which accelerate neurodegeneration[21].

Various research groups have demonstrated that heparin can regulate complement activation at different sites of the cascade by means of a mechanism that is independent of antithrombin III affinity[163,164,165,166,167,168,169,170]. It increases the activity of C1-INH[87,171,172], interferes with the interactions of C4 with C1 and C2, prevents the formation of the C3 amplification loop of the alternative pathway, and attenuates the proinflammatory activities of anaphylatoxin C5a and MAC-dependent hemolysis[53,173]. We have also found that heparin abolishes the ability of Aβ to activate C4 in a dose-dependent manner[48]. The binding sites of heparin and C1q are, respectively, located between residues 12 and 17 (-H-H-Q-K-)[54] and residues 7 and 11 within the N-terminal region of Aβ[160], and so the heparin-induced prevention of C4 activation is also in line with the hypothesis that this drug may sterically interfere with the binding of C1q to Aβ.

## The Contact/Kinin System

A further mechanism of activating the complement cascade is the activation of the contact/kinin system, which occurs in a C1q-independent manner and leads to the generation of kinins[174,175,176,177].

The contact system consists of zymogens factor XII (fXII), factor XI (fXI), and prekallikrein (PK), and the nonenzymatic high-molecular-weight kininogen cofactor (HK)[178,179,180,181,182,183]. fXII self-activates (fXIIa) as a result of contact with a variety of artificial or biologically negative surfaces (contact activation), which leads to blood coagulation and the activation of the inflammatory kallikrein/kinin and complement systems. The fXIIa activation of fXI initiates a series of proteolytic reactions that lead to the thrombin generation preceding clot formation, whereas the fXIIa-dependent activation of PK forms kallikrein, which reciprocally activates more fXII and releases bradykinin from HK. fXIIa then activates the macromolecule complex of the first component of complement, thus leading to the activation of the classical complement system; kallikrein also directly activates complement components C3 and C5[178,184]. Alternatively, increased fibrinolytic activity may activate early complement factors as plasmin can activate C1s *in vitro* and *in vivo*[185,186,187].

Most of the biological surfaces that activate fXII are expressed in disease states, and investigators have long searched for the physiological activators of fXII and its role *in vivo*. Maas et al. recently showed that the misfolded protein aggregates produced during systemic amyloidosis allow plasma fXIIa and PK activation, and the increased formation of kallikrein-C1-INH complexes, without fXI activation and coagulation. This study described a novel biological surface for fXII activation and activity, which initiates inflammatory events independently of hemostasis[188]. We previously demonstrated that HK, a marker of contact system activation, is massively cleaved in the CSF, but not in the plasma of AD patients, which suggests that system activation is a characteristic of the AD brain[94]. Region 1-11 of Aβ is critical for contact system activation and heparin can prevent the Aβ-dependent cleavage of HK in a dose-dependent manner[47]. The unique heparin binding site on Aβ is located between residues 12 and 17 (-H-H-Q-K-) and this could have sterically hindered the negatively charged residues within region 1-11[95].

There is also evidence that inhibiting thrombin activity may be a useful effect of heparin in AD. Thrombin activates platelets, a major source of APP and Aβ release into the bloodstream[189,190], and thrombin receptors on activated platelets can trigger complement activation in the fluid phase. Furthermore, the accumulation of thrombin in neurofibrillary tangles supports the hypothesis that it may also be involved in tau proteolysis[191].

An experimental model of AD recently revealed a significant increase in the density of both kinin receptors (B1 and B2) in the brains of rats after the intracerebroventricular infusion of Aβ[192]. Tissue plasminogen factor (tPA), the primary activator of plasminogen to plasmin in the brain, colocalized with the Aβ plaques, but tPA protein levels were the same as in age-matched control brain tissue. However, as shown for the first time by Fabbro et al., tPA activity in the AD brain is greatly reduced in comparison with age-matched control brain tissue and this may be responsible for the diminished levels of plasmin in AD[193]. Plasmin readily cleaves both fibrillar and monomeric Aβ, thus making it an attractive candidate for Aβ clearance.

Despite these several lines of evidence supporting the hypothesis that the contact system may be involved in AD, it remains to be ascertained whether the functional disturbance of neurons or glial cells is at least partially attributable to the generation of bradykinin induced by contact/kinin system activation. In any case, the activation of fXII and PK leads to the generation of enzymes that react with C1-INH to form fXIIa-C1-INH and kallikrein-C1-INH complexes. *In situ* hybridization has revealed that only neurons express C1-INH mRNA in brain areas with neuritic plaques and activated glial cells, and not the cells (such as astrocytes) that usually produce the complex. Defective synthesis combined with an increased rate of consumption and up-regulated production of complement factor may therefore lead to a functional deficiency in C1-INH, and this local deficiency may facilitate the activation of complement in the affected areas of the AD brain[194].

In clinical terms, heparin-induced potentiation of C1-INH activity may be interesting in AD[195,196]. The presence of a single, major, positively charged region on the contact surface of C1-INH has led to consideration of the simplest sufficient mechanism of GAGs potentiation of C1-INH: charge neutralization. Heparin-like GAGs regulate the catalytic activity of fXIa during interactions with the macromolecular substrate and inhibitors such as the serpins antithrombin (AT) and C1-INH. The results obtained by Yang et al. suggest that basic residues of fXIa form a heparin-binding site, and that the accelerating effect of heparin on the inhibition of fXIa by AT or C1-INH may be mediated by charge neutralization and/or allosteric mechanisms that overcome the repulsive inhibitory interactions of serpins with these basic residues[87].

## HEPARIN AND APOLIPOPROTEIN E

ApoE is a constituent of many lipoproteins that transport lipids between cells throughout the circulatory system[197]. Three common isoforms are expressed in humans: ApoE$\varepsilon_2$, ApoE$\varepsilon_3$, and ApoE$\varepsilon_4$, all of which are products of alleles at a single gene locus. In the brain, ApoE is primarily produced by glial cells and its receptors are abundantly expressed by neurons. By accelerating Aβ aggregation towards mature fibril formation, (human) ApoE prevents the formation of toxic Aβ intermediates such as oligomers and protofibrils, and may protect against the development of AD by suppressing the inflammatory reactions associated with AD lesions[14]. In addition to inducing conformational changes in Aβ, ApoE facilitates Aβ clearance from the brain by acting as an Aβ transporter across the blood-brain barrier (BBB): both the ApoE isoform and the ApoE lipidation state affect Aβ clearance. ApoE$\varepsilon_4$ forms a less stable complex with Aβ than ApoE$\varepsilon_3$ or ApoE$\varepsilon_2$, thus reducing Aβ transport efficiency across the BBB and more efficiently enhancing Aβ aggregation than ApoE$\varepsilon_3$, which also inhibits clearance[21]. In a recent study, Deane et al. showed that Aβ binding to ApoE$\varepsilon_4$ redirected the rapid clearance of unbound Aβ40 and Aβ42 from LDL receptor-related protein-1 (LRP1) to the VLDL receptor (VLDLR), which has a substantially slower endocytotic rate than LRP1 and slowly clears ApoE$\varepsilon_4$ and Aβ-ApoE$\varepsilon_4$ complexes[198]. In addition to colocalizing with Aβ in AD brains, ApoE is also found in neurons containing neurofibrillary tangles, where it interacts directly with tau protein. Furthermore, ApoE has an isoform-dependent effect on tau phosphorylation: ApoE$\varepsilon_3$ binds to tau *in vitro*, but ApoE$\varepsilon_4$ does not. An ApoE$\varepsilon_4$-dependent increase in phosphorylated tau has also been observed.

The ApoE genotype is the only established genetic risk factor for late-onset sporadic AD[199]. The ApoE$\varepsilon_4$ genotype is more frequent in sporadic and familial late-onset AD, occurring in about 52% of all cases of familial AD as against 16% of healthy controls[200]. In elderly people without dementia, the ApoE$\varepsilon_4$ allele is associated with a more than twofold greater risk for developing AD[201]. In subjects with MCI, the presence of the ApoE$\varepsilon_4$ genotype is a strong predictor of progression to AD[202,203]. Various lines of research suggest that the ApoE$\varepsilon_4$ allele may be associated with AD by means of its role in the development of amyloid disease. ApoE$\varepsilon_4$ has been related to increased Aβ production and deposition and, as discussed above, BACE-1 is pivotal for Aβ generation, and a new study has shown an association between the ApoE$\varepsilon_4$ genotype and BACE-1 activity measured in the CSF of AD and MCI subjects[92].

There is evidence that the highly positively charged APOE$\varepsilon_4$ lysine (Lys) and arginine (Arg) residues (Arg-Lys-Leu[leucine]-Lys-Arg) can bind Aβ monomers, which are unable to polymerize and assemble into large aggregates**,** thus accelerating fibril formation and maintaining fibril stability. An early study[204] found that heparin inhibits the effects of Lys and Arg by binding to positively charged ApoE$\varepsilon_4$ residues, thus minimizing the effects of ApoE$\varepsilon_4$ and having a beneficial effect on the pathogenesis of AD[205,206,207,208,209,210].

## CONCLUSION

AD is characterized by a decline in intellectual function that is severe enough to interfere with normal daily activities and social relationships. Its neuropathological hallmarks are amyloid deposition in senile plaques, neurofibrillary tangles, and neuronal cell loss in a number of cortical and subcortical regions. Although the exact pathogenesis of AD has not yet been fully defined, various pharmacological strategies for preventing and treating it are being actively investigated, some of which involve the interactions of some compounds with the production and aggregation of Aβ. Moreover, a new drug has targeted the molecular events involved in AD, such as the cytotoxicity of Aβ and its ability to trigger a robust local inflammatory reaction.

Heparin has been proposed as a promising agent because of its multiple effects on the pathogenesis of AD, including its potential competitive interactions with proteoglycans, vascular effects, interactions with serpins, and anti-inflammatory activity. Given the relationships between these different mechanisms, the pleiotropic effects of heparin and heparin-related compounds may have greater therapeutic potential than compounds directed against a single target. As heparin remains one of the most important anticoagulant drugs in clinical practice, a greater understanding of the structure-activity relationships between its anticoagulant effects and anti-inflammatory mechanisms has aroused increased interest in heparin and its derivatives as a new treatment of inflammatory disease. The therapeutic use of GAGs in the brain is limited by the extent to which the different compounds penetrate the BBB and, therefore, their molecular weight. Low-molecular-weight GAGs, including low-molecular-weight heparin (LMWH), may have more therapeutic value than molecularly heavier substances. Two important findings have provided a basis for the development of heparin oligosaccharides in this area: heparin's inhibition of inflammatory responses is independent of its anticoagulant activity, and heparin oligosaccharides have similar or even better anti-inflammatory effects than heparin itself[55,64]. Dudas et al.[211,212] recently demonstrated that some similar low-molecular-weight GAGs (certoparin, C3, C6) are capable of crossing the BBB and preventing Aβ-induced conformational changes in tau and reactive astrocytosis. Our previous *in vitro* studies demonstrated that enoxaparin, a LMWH, is as capable as heparin of attenuating the neurotoxicity and proinflammatory activity of Aβ. We also found that prophylactic treatment with a clinically relevant dose of enoxaparin reduces reactive astrocytosis, and the deposition and total brain concentration of Aβ in a mouse model of AD (APP23 mice), and the absence of an inflammatory reaction and brain hemorrhaging suggests that the long-term treatment was well tolerated[49]. We are currently conducting a preclinical study of a prophylactic dose of enoxaparin and the preliminary 3-month results indicate that it is well tolerated by AD patients.

This paper summarizes the possible beneficial effects of heparins that are distinct from their well-known anticoagulant activity, but more experimental data are needed in order to define the relationship between their structure and antiamyloid effects (Fig. 3).

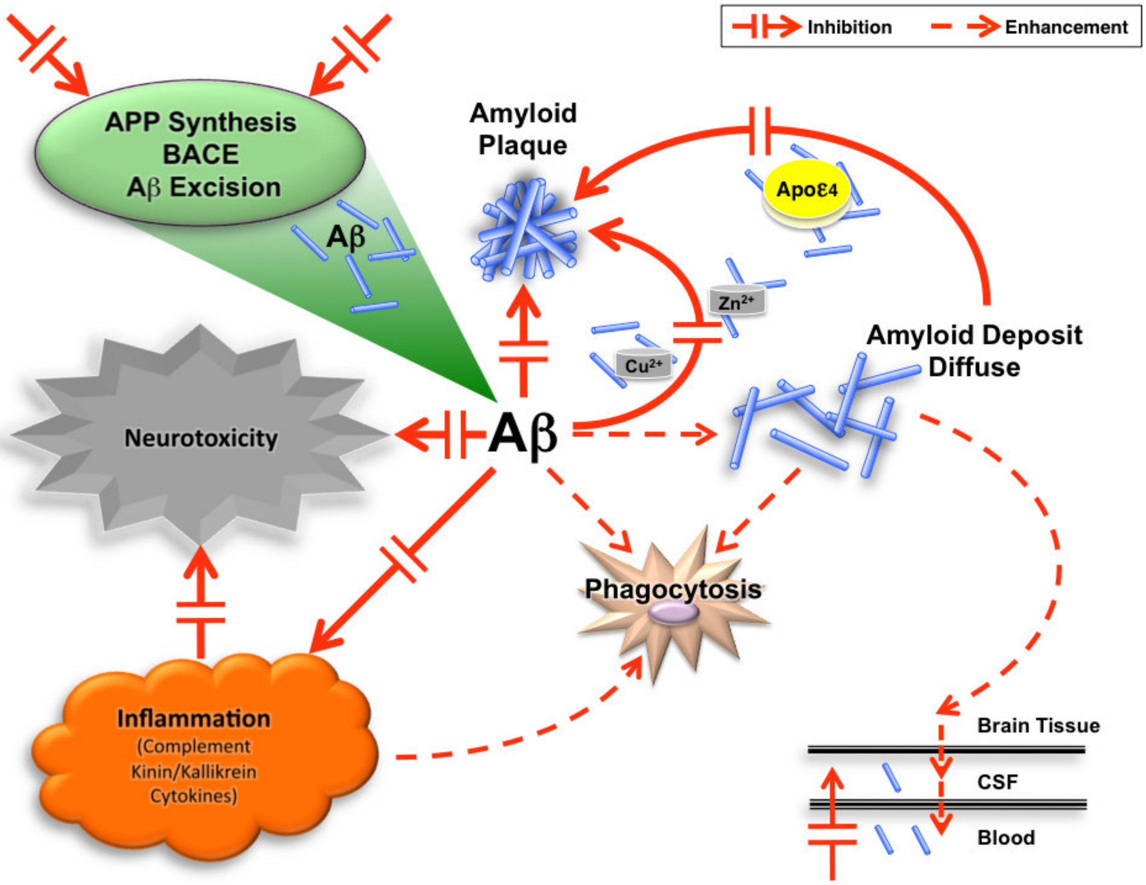

**FIGURE 3.** Possible protective mechanisms of heparins in AD pathology: reduction of Aβ generation by an action on APP processing or metabolism, prevention of Aβ aggregation/deposition in senile plaques, inhibition of Aβ-driven toxic effects (inflammation, neurotoxicity), reduction of the inflammatory response (complement, kinin system), facilitation of Aβ removal from the brain compartment.
